# Curettage—Its Indications and Technique

**Published:** 1903-01

**Authors:** Monroe Smith

**Affiliations:** Atlanta, Ga.


ATLANTA

### Journal-Record of Medicine.

Successor to Atlanta Medical and Surgical Journal, Established 1855, and Southern Medical Record, Established 1870.

VOL. IV. JANUARY, 1903. No. 10.

BERNARD WOLFF, M.D., EDITOR, Nos. 319-20 Prudential.

Published Monthly.

M. B. HUTCHINS, M.D., BUSINESS MANAGER, No. 64 Marietta St.

### ORIGINAL COMMUNICATIONS.

### CURETTAGE—ITS INDICATIONS AND TECHNIQUE.

BY MONROE SMITH, M.D.,

ATLANTA, GA.

Uterine curettage is perhaps the most frequently performed of all surgical operations, and yet it is one which is frequently neglected when it should be done ; and on the other hand, very often improperly and incompletely performed. It is one of the simplest of all surgical procedures, if thoroughly and properly performed, but on the other hand, if done otherwise than properly, may become a very serious surgical operation.

The usual indications of curettage are uterine hemorrages from various causes. Anything acting as a foreign body within the uterus, such as retained membranes from an incomplete abortion, or a retention of fragments of the placenta after confinement, the presence of fungosities, etc., will set up uterine hemorrhage, and is a most imperative indication of curettage. As another very frequent cause of uterine hemorrhage, and therefore an indication for curettage, may be mentioned uterine polypus. Chronic endometritis is also another indication for curettage, as it affords the safest and, in fact, the only cure for that condition. Puerperal infection, as represented by an irregular temperature, usually preceded by a chill, with or without a foul lochia, always calls for a careful and thorough curettage.

The most frequent indication for curettage in my practice I have found to be incomplete abortion. Practicing medicine in Atlanta, a place of more than 100,000 inhabitants, where the requirements of society are very strenuous, which produces a loathing for offspring on account of close confinement at home for months before and after the confinement, it is not at all surprising that so many of our women, when they find that they are pregnant, seek relief by the various methods and means usually employed to bring about an abortion. It is frequently the case, when they cannot get some physician to do it for them, that they will resort to a sound, a hairpin, or a knitting-needle, and in some cases I have known them to employ a lead pencil when nothing better was available. This instrument they are taught, by their friends, to introduce into the womb, where it breaks up the membranes, frequently producing sepsis of very grave character, uterine hemorrhage, etc., for which condition, when the doctor is called, there is nothing to be done but to anesthetize the patient, and under strict asepsis dilate and empty the uterus.

However, it has been my experience, in abortion before the third month, and sometimes at a later period, which is brought about from various causes independent of the woman's will or wishes in the matter, from a retro-displaced uterus or from any other cause, that as a rule the miscarriage is incomplete, and consequently requires a curettage for its completion. It is my practice in every case of inevitable abortion, to which I am called, to at once empty the uterus. I am more heroic now in my practice with these cases than I once was, for the reason that I have seen more than one case of inevitable abortion, in which the emptying of the uterus had been postponed for a few hours for a more convenient time, or maybe owing to a desire to please the patient and give nature an opportunity of throwing off its burden, that while the doctor was absent, or probably could not be readily procured, hemorrhage of a severe and alarming character set up, and, when help was secured, the patient was exsanguinated and life was lost. The uterus can be safely and very quickly emptied in this condition at any and all hours of the night, and I dc not believe that it is a fair practice to the patient, much less a safe one, to postpone these cases till daylight, though the vagina may be fairly tamponed with cotton, for that frequently does no more good than to simply absorb the blood, which continues to pour from the uterus, until the cotton is thoroughly soaked, thus deceiving the doctor into a sense of safety, thinking that the hemorrhage is controlled when in reality it is continuing as though the vagina was not packed.

The same remarks as the above hold true in retention of fragments of the placenta, as in cases where the membranes are left behind, or in cases of adherent placenta, where the placenta separates within its own walls, leaving a part of it behind. In every case of puerperal sepsis it is my practice, when I have formed a positive diagnosis of the condition, to at once explore the uterus carefully and thoroughly to assure myself that there are no fragments of the placenta or membranes left behind which may be responsible for the condition, and I do this before the patient has been depleted and exhausted from continued high fever produced by the septic condition.

In almost every case of irregular and profuse hemorrhage from the uterus in the non-pregnant condition, whether there are or are not fibroid growths of the uterus, you will find that a good, careful curettage will remove more or less of the fungous growths, add, if thoroughly scraped away, and the uterus douched the hemorrhage will cease for at least several weeks.

The technique of curettage is simple, and is performed as follows by myself: The patient being anesthetized is placed in the lithotomy position, the parts thoroughly disinfected, as well as hands, instruments, etc. I first ascertain by bimanual examination the size and position of the uterus, when I introduce into the vagina Garrigue's Weight Speculum, which retracts the perineum perfectly by its own weight. I then grasp the anterior lip of the uterus with a Volsella forceps. The uterus is pulled down to within an easy working distance. The uterine sound is next introduced to get the depth, and to assure myself of the exact uterine position. Next I take my uterine dilaotr, and introduce it into and through the internal os, which, when the handles are squeezed, stretches the cervical canal open. The dilator is turned from side to side, and the dilating continued until sufficiently completed. Next, if in case of fungosities, I use the small sharp curette, with which I thoroughly scrape the entire uterine canal. I next use the irrigating curette spoon, and, while the water is flowing freely in the uterine canal, I carefully scrape all fragments to the os, which are from there washed out. In case of incomplete abortion, the bulk of the membranes are removed by a pair of placenta forceps, or any simple forceps which may be convenient, when I then employ a large curette, with mouse teeth, with which I carefully explore the entire uterus, and on account of the rough condition of the curette all the membranes are easily and quickly removed. The same remarks also apply to retained membranes, etc., from confinement.

In all cases, where the dilatation is sufficient to admit it, before I consider the uterus completely emptied, I pass my finger into the uterus to confirm that fact. In fact, in a great many cases the finger makes one of the safest and most efficient instruments for curettage.

In all cases of curettage, when I think the uterus is pretty thoroughly emptied—perhaps nothing remaining but a few small fragments—I then thoroughly irrigate it with a double-strength salt solution. I never use anything as an intra-uterine douche except salt solution, for the reason that no one can tell, however careful you may be, when, without any pressure and seemingly without any provocation, the curette will at once be found to have slipped through the uterine wall and be within the abdominal cavity. In such cases you can readily imagine your dilemma, if you are using as an irrigating fluid bichloride solution, carbolic acid solution, or any other of the various poisonous drugs which are recommended and are almost—I am sorry to say—universally used by the less experienced members of the profession. I have curetted a great many wombs and under almost all conditions. I feel assured that I do it as carefully and as effectually as any one can, and yet it has been my experience and my dismay to have punctured a number of wombs. I do not believe that it is dangerous to use those fluids in intra-uterine douche except in cases where the uterus is punctured and the fluid consequently thrown into the abdominal cavity, for I do not believe that the uterus itself would absorb a sufficient quantity of such fluids to produce any systemic effects.

Puncture of the womb, except in septic cases, if you are using salt solution at the time of the puncture, is not of any serious concern, and should not interrupt the completion of the curettage, for from the general symptoms, outside of possibly a little griping pain in the abdomen for a few hours, there will be no other symptom to indicate that the uterus has been disturbed any further than in the usual curettage. In those cases, however, if there be no other indication, when the curettage is completed, I pass a small strip of iodoform gauze up into the womb, which is left in place forty-eight hours to insure thorough uterine drainage.

The contraindications of the curettage are not many. The physical condition of the patient first is to be considered, but in cases of uterine hemorrhage I would do a curettage as a means of arresting hemorrhage in very extreme cases. Pelvic cellulitis, metritis, salpingitis, etc., I would consider as contraindications to curettage. The dangers of curettage are usually given as peritonitis, pelvic cellulitis, atresia of uterine canal, hemorrhage coming on immediately or perhaps hours after the operation, puncture of the uterus, etc. Peritonitis and pelvic cellulitis are two conditions which I have never had follow the operation in my hands. Hemorrhage may not be feared, except in rare cases of incomplete abortion and curettage done during the first few days subsequent to confinement, and then the hemorrhage comes on at once and can be controlled by packing the uterus with iodoform gauze. Atresia of the uterine canal is a condition I have had twice in my experience, which followed a thorough curetting of the uterus for endometritis, in which cases I used no packing, and also no sounds were introduced into the uterus for several weeks after the operation. Since this experience, in every case of curettage of the uterus for endometritis or fungosities, where the uterus is thoroughly explored and the endometrium thoroughly scraped, I make it a rule to pass a strip of iodoform gauze into the uterus, and leave it there for forty-eight hours, when it is removed. With this treatment I do not believe you will have uterine atresia.

The after-treatment of these cases will depend upon the cause for which the curettage is done. In cases of endometritis or fungosities I remove the gauze, as above indicated, in forty-eight hours, and then have the patient given some antiseptic douche once or twice daily for ten days or two weeks thereafter, with aseptic vulvar pads applied, and the patient kept in bed five or six days, or perhaps longer. In case of incomplete abortion, if there is no hemorrhage requiring tamponing to control, I leave no gauze within the uterus; use simply aseptic pads to the vulva for a number of days, when, if there be an indication, vaginal douches are given. Where there is a good deal of traumatism incident to the curettage, it is my custom to have an ice bag applied over the fundus of the uterus for twenty-four or forty-eight hours, according to the indications.

